# Assessing the Impact of Oil Types and Grades on Tocopherol and Tocotrienol Contents in Vegetable Oils with Chemometric Methods

**DOI:** 10.3390/molecules25215076

**Published:** 2020-11-01

**Authors:** Yunqi Wen, Lili Xu, Changhu Xue, Xiaoming Jiang, Zihao Wei

**Affiliations:** College of Food Science and Engineering, Ocean University of China, Qingdao 266003, China; wenyq0715@163.com (Y.W.); xulili8339@163.com (L.X.); oucxuech@163.com (C.X.)

**Keywords:** tocopherols, tocotrienols, oil types, oil grades, vegetable oils, chemometric

## Abstract

The consumption of vegetable oil is an important way for the body to obtain tocols. However, the impact of oil types and grades on the tocopherol and tocotrienol contents in vegetable oils is unclear. In this study, nine types of traditional edible oils and ten types of self-produced new types of vegetable oil were used to analyze eight kinds of tocols. The results showed that the oil types exerted a great impact on the tocol content of traditional edible oils. Soybean oils, corn oils, and rapeseed oils all could be well distinguished from sunflower oils. Both sunflower oils and cotton seed oils showed major differences from camellia oils as well as sesame oils. Among them, rice bran oils contained the most abundant types of tocols. New types of oil, especially sacha inchi oil, have provided a new approach to obtaining oils with a high tocol content. Oil refinement leads to the loss of tocols in vegetable oil, and the degree of oil refinement determines the oil grade. However, the oil grade could not imply the final tocol content in oil from market. This study could be beneficial for the oil industry and dietary nutrition.

## 1. Introduction

**Figure 1:**
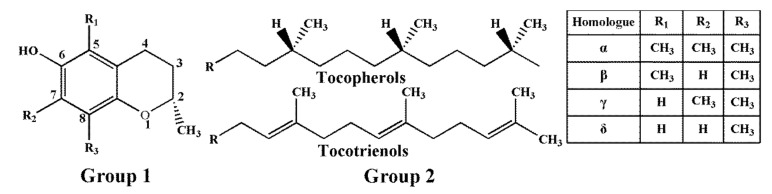
Chemical structures of T and T3. Group 2 (16-carbon phytyl side chain) is attached to group 1 (chromanol ring) at position 2.

Tocopherols and tocotrienols, collectively referred to as tocols or vitamin E, are a family of naturally occurring fat-soluble compounds, which include eight members (α-, β-, γ-, and δ-tocopherol (T); α-, β-, γ-, and δ-tocotrienol (T3)) [1]. As shown in Figure 1, tocols consist of two primary parts: the chromanol ring and the hydrophobic side chain. The hydrophobic side chains of four tocopherols are saturated isoprenoid side chains, and four tocotrienols are isoprenyl side chains with three double bonds [2,3]. The chromanol ring is able to donate a hydrogen atom to reduce free radicals, and the hydrophobic side chain allows the molecule to penetrate into biological membranes [4].

Tocols are well known for their potent antioxidant capacity and are vitally needed in relatively small amounts to maintain life and promote good health [1]. The main biochemical function of tocopherols is considered as the protection of polyunsaturated fatty acids against peroxidation, and the activity of tocopherols may depend on the ratio of total tocopherols to polyunsaturated fatty acids [5]. Tocols are also used for the extension of shelf life and the stabilization of food. Tocopherols play an important role in preventing the oxidation of almond lipids, resulting in increasing the possibility of lengthening kernel storage [5,6,7]. Recently, the cosmetic industry and clinical dermatology have paid more attention to tocols due to their photoprotection and antioxidant properties [8]. Tocols can limit lipid peroxidation in cell membranes and scavenging reactive oxygen species and protect tissues from consequent oxidative damage. Thus, supra-nutritional doses of tocols have been reported for cardiovascular diseases, cancer, chronic inflammation, Alzheimer’s disease, and Parkinson’s disease [9]. Tocotrienols are reported to be superior to tocopherols in the prevention and treatment of major chronic diseases, as their unsaturated chain facilitating cell penetration is highly antioxidative [10]. Among tocopherols, α-T is the only form incorporated in very low-density lipoproteins (VLDL) by means of a specific liver protein, leading to greater biological activity [11]. However, γ-T, compared with α-T, is more capable of trapping electrophiles and reactive nitrogen species (RNS) during inflammation [12].

For human nutrition, diet is the major source of tocol intake. Especially when compared with animal-derived foods, edible plant-derived products are the major source of tocols, such as oil seeds; green parts of plants; whole grains; nuts; and vegetable oils of wheat germen, sunflower, corn, as well as olives [2,13,14,15,16]. Vegetable oils, quintessential lipid foods in the human diet, are considered very suitable for the enrichment of tocopherols due to the lipophilicity of tocopherols [17]. Besides this, the content of tocols in oil is considered an important indicator to reflect the oil quality. At present, many varieties of traditional edible oils have appeared on the market. Additionally, some new types of vegetable oil have drawn more attention since they are identified as new resource foods, such as sumac fruit oil, sacha inchi oil, swida wilsoniana oil, and so on. The tocol content may vary greatly between different types of vegetable oil. However, the relationship between tocol content and the type of oil has not been revealed. Vegetable oils are usually classified into different grades according to their values of quality and sanitation indexes (such as acid values, peroxide value, iodine value, etc.). The grade of oil is closely related to the degree of oil refinement. For instance, the refining degree of first-grade oil is higher than that of fourth-grade oil, which may imply that the vitamin E loss of the first-grade oil is higher than that of the fourth-grade oil. However, the specific correspondence between the oil grade and vitamin E content is rarely reported.

In recent years, many studies have investigated the tocols of edible oils. Most of them focus on extraction methods, chromatographic separation, determination methods, and the nutritional characteristics of tocols [4,18,19,20,21]. Therefore, in terms of tocol determination, the amount of analyzed oil samples is limited. Moreover, some of these studies deal with only part of eight tocols, which results in an incomplete understanding of tocol distribution in traditional oils. Recently, many studies with respect to new types of oil have been carried out. They usually focus on the preparation methods, physicochemical properties, antioxidant properties, nutritional properties, and applications of oils [22,23,24,25,26,27,28,29,30,31]. As a result, the information about tocols in new types of vegetable oil is severely scarce. To achieve a comprehensive understanding of the relationship between the tocol distribution and different species as well as the grades of edible oils, 9 types of 146 traditional edible oil samples and 10 new types of vegetable oil were selected for the evaluation using chemometric methods. Traditional edible oils were directly sampled from factories, and new types of vegetable oil were self-produced in our lab (including extraction and refining processes) by the use of 10 types of oil seeds purchased from markets. This study will be beneficial for people selecting suitable edible oils for dietary nutrition. Besides this, it can provide strong support for the development of new types of oil and corresponding oil seed.

## 2. Results and Discussion

### 2.1. Validation of Analytical Method

Six factors were evaluated to characterize the method of tocol determination and demonstrate its suitability. The results are summarized in Table S1. The correlation coefficients were above 0.9987, indicating the excellent linearity of the calibration curves. All of the tocols exhibited satisfactory recoveries at the three spiking levels, and the average recoveries ranged from 84.3% to 109.7%. Each vegetable oil sample was analyzed six times in one day for the intraday precision, and all of the tocols presented in the range of 1.1–6.7%. For the interday precision, vegetable oil samples were analyzed three times during three continuous days. The range of interday precision was 2.4–7.4%. All the precision values were below the accepted limit of 10%, indicating that the applied analytical method was suitable for the current research. The LOD and LOQ was in the range of 0.08–0.14 and 0.28–0.48 mg/kg, respectively.

### 2.2. Influence of Oil Types on Tocopherol and Tocotrienol Contents in Vegetable Oils

#### 2.2.1. Influence of Oil Types on the Tocopherol and Tocotrienol Contents in First-Grade Traditional Edible Oils

First-grade edible oil has the largest consumption among all grades of vegetable oils. Nine types of first-grade traditional edible oils were analyzed in terms of their tocol contents in our study, and the results are summarized in Table 1. Rice bran oils contained seven kinds of tocols. Both corn oils and cottonseed oils contained four kinds of tocols. Camellia oils included only α-T and γ-T, and sesame oils contained only γ-T. 

**Figure 2:**
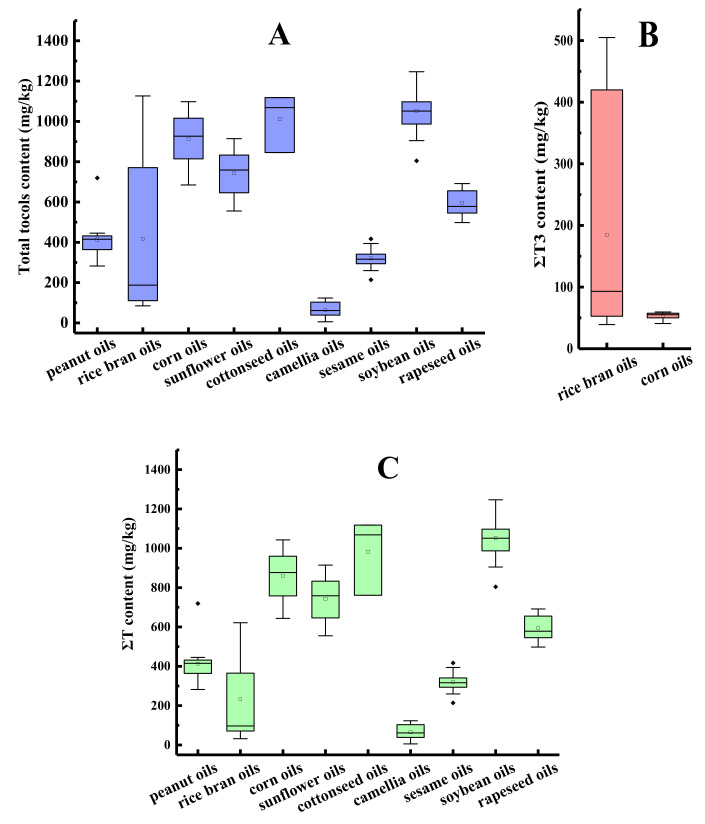
The tocol contents in different species of first-grade traditional edible oils (mg/kg). (**A**) Total tocol content; (**B**) ΣT3 content; (**C**) ΣT content.

The ΣT content, ΣT3 content, and total tocol content in different species of first-grade traditional edible oils are shown in Figure 2. Horizontal inner lines in the boxes are the mean values. As depicted in Figure 2A, the average contents of the total tocols were compared and listed in the following order: soybean oil > cottonseed oil > corn oil > sunflower oil > rapeseed oil > rice bran oil > peanut oil > sesame oil > camellia oil. The average content of total tocols in the first-grade traditional edible oils ranged from 65.7 mg/kg of the camellia oil samples (*n* = 6) to 1052.6 mg/kg of the soybean oil samples (*n* = 23). Different rice bran oil samples showed large differences in the total tocol content, with the coefficient of variation (CV) presenting as 95.3%, and the highest total tocol content of the rice bran oil sample was 13.3 times higher than that of the lowest. The probable reason for the variation in the tocol contents may be the different cultivars of crops, growing environments, methods for oil extraction, or analytical methods used to detect the tocols [32,33,34].

As shown in Figure 2C, the soybean oil samples had the maximum average content of tocopherols (1052.6 mg/kg) and camellia oil samples had the minimum average content of tocopherols (65.7 mg/kg). The tocopherol contents of sunflower oil were 891 mg/kg [35] or in the range between 562.8 and 1872.8 mg/kg [36], which was also consistent with our result. Tocotrienols were much less prevalent than tocopherols in vegetable oils [37]. Among the nine types of traditional edible oils in our study, tocotrienols were only found in rice bran oils, corn oils, and some of cottonseed oils, with average contents of 184.9, 53.4 (Figure 2B), and 28.2 mg/kg, respectively. The tocotrienol content accounted for 44.2%, 5.8%, and 2.8% of the total tocol content, respectively.

Based on previous studies about the tocol contents in vegetable oils (Table 2), the soybean oil contained 958 mg/kg of total tocols and peanut oil contained 367 mg/kg of total tocols [1]. The tocol contents of soybean oil and sunflower oil were 999 and 768 mg/kg, respectively, as reported by Adhikari [38]. The tocol contents of soybean oil and rapeseed oil were 1026.1 and 649.1 mg/kg, respectively [20]. Besides this, the soybean oil, corn oil, rapeseed oil, and rice bran oil contained 1135.8, 808.3, 555.2 and 867.2 mg/kg of total tocols, respectively [39]. The contents of total tocols in all these vegetable oils mentioned above were within the range of tocol contents provided by our study. Research conducted by Mejean et al. [40] showed that 1886 mg/kg of total tocols was found in soybean oil, which was higher than the maximum content of total tocols (1246.6 mg/kg) in our study. However, the result of peanut oil (containing 259.6 mg/kg of total tocols) was lower than the minimum content of total tocols in our study.

The most widely distributed and biologically active tocol was α-T, and the activities of β-T, γ-T, and δ-T were 30%, 15%, and 3% of the activity of α-T, respectively [2]. Sunflower oils had the maximum average content of α-T (718.4 mg/kg), followed by cottonseed oils and peanut oils. Camellia oils contained the lowest average content of α-T (59.6 mg/kg), and sesame oils contained γ-T only. In accordance with our results, three studies reported that the contents of α-T in sunflower oils were 714 [19], 604.6 [39], and 720 mg/kg [4], respectively. Camellia oils contained 153 mg/kg of α-T [43], which was similar to the maximum content of α-T in our study. Approximately 70% of the tocol intake from food sources in the United States was in the form of γ-T, because Americans have a high intake of soybean oil and other vegetable oils that are rich in γ-T [44]. Soybean oils had the maximum average content of 675.5 mg/kg in our study, followed by corn oils. Camellia oils contained the lowest average content of γ-T (6.2 mg/kg). Studies have stated that soybean oils contain 642.7 [20], 823.1 [39], and 390–690 mg/kg [41] of γ-T, respectively, which was almost in accordance with our results. Neither the camellia oils nor sesame oils contained a detectable amount of δ-T in our study.

**Figure 3:**
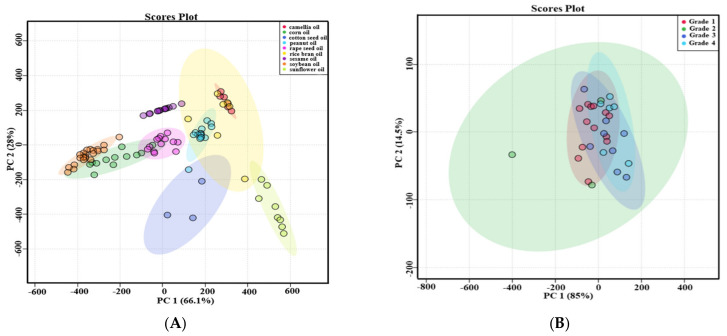
PCA score plots of the selected traditional edible oils. (**A**) Different types of first-grade traditional edible oils; (**B**) different grades of rapeseed oils.

**Figure 4:**
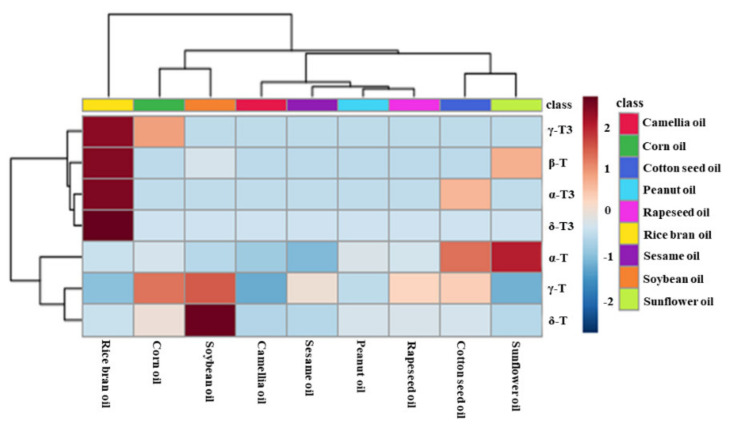
Heatmap explains the different contents of tocopherols and tocotrienols in the first-grade traditional edible oils (red indicates tocols distributed at a high concentration and blue indicates tocols distributed at a low concentration).

Principal component analysis (PCA) was used to evaluate the difference among nine types of first-grade traditional edible oils. Figure 3A showed that the top two principal components (PC1 and PC2) accounted for 66.1% and 28.0% of the total variability, respectively, which represents 94.1% of the total variance. According to the score values of PC1, soybean oils, corn oils, and rapeseed oils could be all well distinguished from sunflower oils. Moreover, sunflower oils and cotton seed oils could be well distinguished from camellia oils and sesame oils according to the score values of PC2. Heat map visualization can directly provide intuitive visualization of the tocol contents of first-grade traditional edible oils. Figure 3 also profiles the similarity among the contents of tocols in first-grade traditional edible oils. Rice bran oil contained a higher content of tocotrienols than the other eight kinds of traditional edible oils, so it was classified separately. Corn oils and soybean oils were classified into the same category, and both of them had a higher content of γ-T. Sunflower oils and cotton seed oils were classified into the same category, and they both had a higher content of α-T. The other four types of edible oils were classified into the same category, as all of them had a lower content of tocols. Figure 4 also shows that tocotrienols and β-T were not commonly found in traditional edible oils.

#### 2.2.2. Influence of Oil Types on Tocopherol and Tocotrienol Contents in New Types of Vegetable Oil

**Figure 5:**
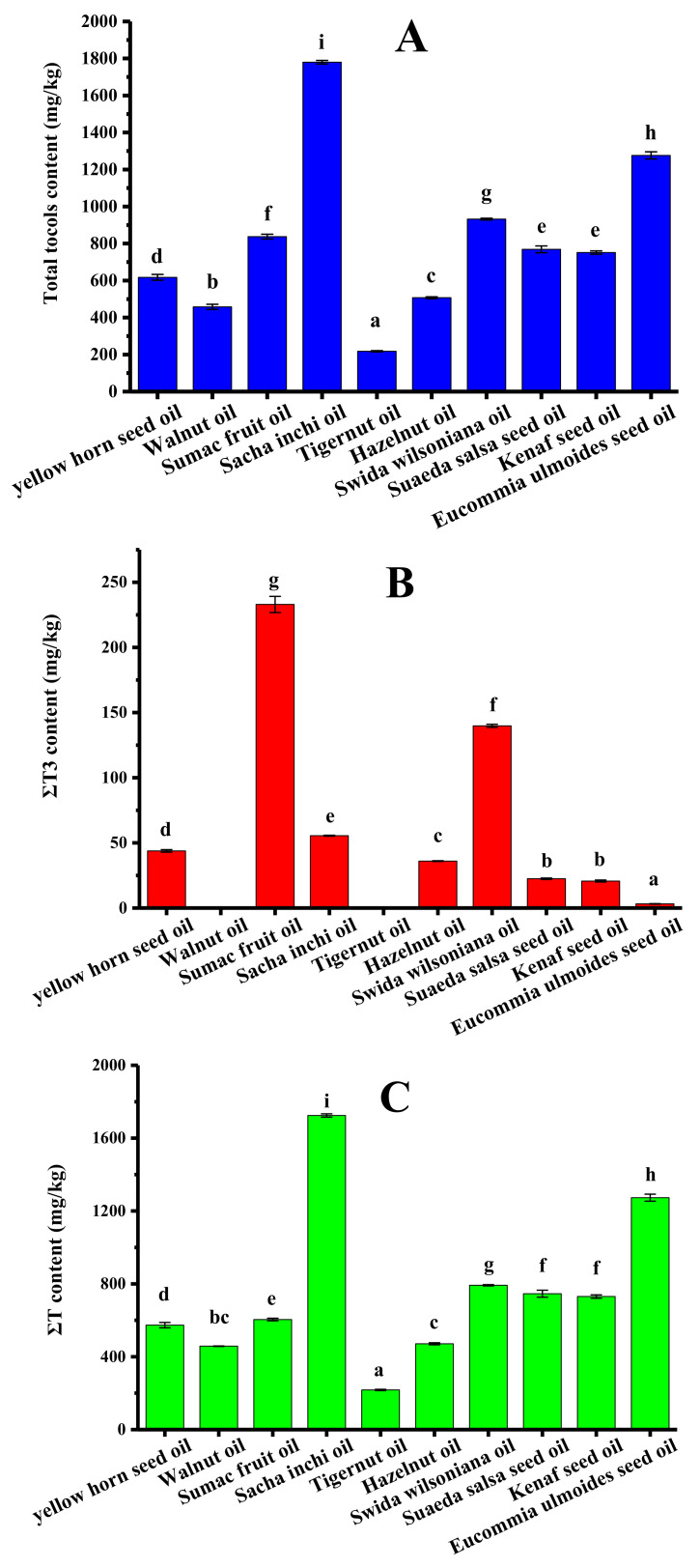
The tocol contents in different new types of vegetable oil (mg/kg). (**A**) Total tocol content; (**B**) ΣT3 content; (**C**) ΣT content (samples with the same letter in a column are not significantly different (*p* < 0.05) *n* = 3).

The tocol contents of ten types of refined new types of vegetable oil were analyzed, and the results are shown in Table 3. Sumac fruit oil contained seven kinds of tocols except for α-T3. Both hazelnut oil and eucommia ulmoides seed oil contained five kinds of tocols. Only two kinds of tocols were detected in tigernut oil and swida wilsoniana oil. It is worth mentioning that only tocopherols existed in walnut oil and tigernut oil. Figure 5 shows the ΣT content, ΣT3 content, and total tocol content in different new types of vegetable oil. The total tocol contents in these vegetable oils ranged from 217.8 (tigernut oil) to 1780.1 mg/kg (sacha inchi oil), and the total tocols contents decreased in the following order (Figure 5A): sacha inchi oil > eucommia ulmoides seed oil > swida wilsoniana oil > sumac fruit oil > suaeda salsa seed oil > kenaf seed oil > yellow horn seed oil > hazelnut oil > walnut oil > tigernut oil. As shown in Figure 5C, sacha inchi oil and tigernut oil obtained the maximum level (1724.6 mg/kg) and the minimum level of tocopherols (217.8 mg/kg), respectively. Figure 5B shows that tocotrienol content ranged from 3.2 mg/kg of eucommia ulmoides seed oil to 233.0 mg/kg of sumac fruit oil. The tocopherol content accounted for 72.2–100.0% of the total tocol content.

**Figure 6:**
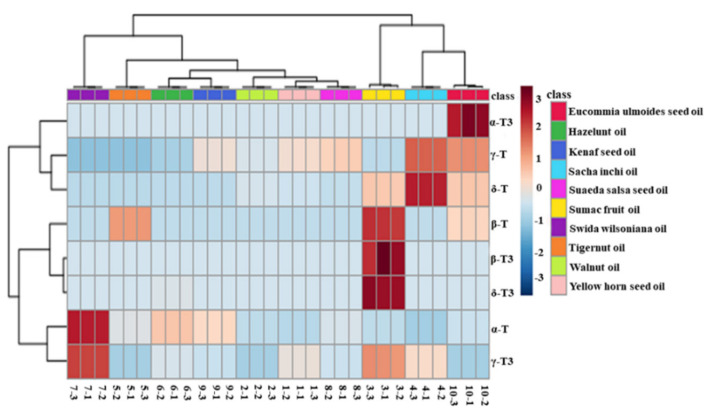
Heatmap explains the different contents of tocopherols and tocotrienols in new types of vegetable oil (red represents tocols that are found at a high content and blue represents tocols that are found at a low content).

Figure 6 provides the intuitive visualization of the tocol contents of new types of vegetable oil. Swida wilsoniana oil was the best source of α-T, with a content of 792.4 mg/kg, followed by hazelnut oil and kenaf seed oil. Sacha inchi oil contained the highest content of γ-T (1016.0 mg/kg), followed by eucommia ulmoides seed oil (851.1 mg/kg). Sacha inchi oil also contained the highest content of δ-T (708.6 mg/kg) among these new types of vegetable oil. Consistent with earlier published data [45], the tocopherol content in hazelnut oil was 572.27 mg/kg. Additionally, eucommia ulmoides seed oil, sumac fruit oil, and kenaf seed oil have been reported to contain 1218.24, 876.95, and 531.5 mg/kg of total tocols, respectively, which are all similar to the values found in our study [30,42].

Figure 6 also profiles the similarity among the contents of tocols in these new types of vegetable oil. Eucommia ulmoides seed oil, sacha inchi oil, and sumac fruit oil were classified into the same category, as they contained more species and higher contents of tocols. Furthermore, swida wilsoniana oil and six other new types of vegetable oil could be classified into the two categories because of high contents of α-T and γ-T3 were found in swida wilsoniana oil. For individual tocol, α-T3, γ-T, and δ-T showed similarities. The other five kinds of tocols could be further divided into two groups, and α-T and γ-T3 belonged to the same group. Therefore, the distribution of tocopherols in new types of vegetable oil was not significantly different from that of tocotrienols.

It can be seen that there may be significant differences in the tocol content of different types of traditional edible oils as well as new types of vegetable oil. At the same time, it should be noted that, even for the same variety of oils, there are also differences in the tocol content (according to the results of the tocol contents of traditional edible oils). This situation may be caused by different growth environments of oil crops, differences in oil extraction methods and so on. Compared with traditional edible oils, the total tocol content in sacha inchi oil and eucommia seed oil was higher. Meanwhile, the contents of α-T in swida wilsoniana oil and γ-T and δ-T in sacha inchi oil were also higher than those of most traditional edible oils. Therefore, it can be speculated that the development of new types of oil is a new approach to obtaining oils with a higher tocol content, which also supports the notion that new types of vegetable oil exhibit a high development value.

### 2.3. Influence of Oil Grades on Tocopherol and Tocotrienol Contents in Vegetable Oils

#### 2.3.1. Influence of Refining on Tocopherol and Tocotrienol Contents in Vegetable Oils

**Figure 7:**
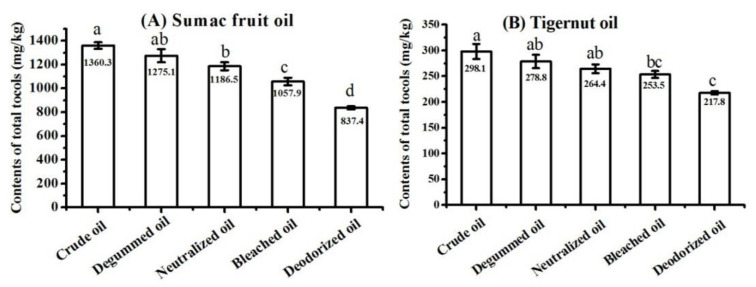
Content of total tocols in sumac fruit oil (**A**) and tigernut oil (**B**) at different refining stages (samples with the same letter in a column are not significantly different (*p* < 0.05) *n* = 3).

The crude oils, degummed oils, neutralized oils, bleached oils, and deodorized oils (also referred to as refined oils) of sumac fruit and tigernut were collected and analyzed, respectively. The total tocol contents in these vegetable oils from different technological stages are presented in Figure 7. The crude oil of sumac fruit contained 1360.3 mg/kg total tocols, which was higher than that of vegetable oils from other four refining stages. The deodorized oils of sumac fruit had the lowest content of total tocols (837.4 mg/kg). It was worth mentioning that the total tocol contents in sumac fruit oils continuously decreased during the refining processes, which also occurred in the refining processes of tigernut oil. The loss rates of total tocols with regard to the four refining stages of sumac fruit oil were calculated, showing as 6.3%, 6.9%, 10.8%, and 20.8%, respectively. The loss rates for tigernut oil were 6.5%, 5.2%, 4.2%, and 14.1%, respectively. The greatest loss of total tocols occurred in the deodorization process. Similar results were reported in previous studies. The study on the refining process of sunflower oil revealed that the tocol contents would reduce during refining processes [46], and the contents of tocopherol in evening primrose oil were lowered by 23.5% during the chemical refining process [47]. Besides this, one report has proposed that the total tocopherol contents gradually decrease during all the refining processes in all oil types, and these significant losses occur especially during the deodorizing stage [48]. Medina-Juáreza et al. [49] pointed out that the proper operational conditions were important during the refining process in order to reduce the loss of tocols. In the degumming process, colloidal impurities have a certain adsorption capacity, and tocols may enter the oil feet after being adsorbed by colloidal impurities, resulting in a loss of tocols in the oil. In the neutralization process, the soapstock produced by the neutralization reaction also has a certain adsorption capacity, which also may lead to a loss of tocols. The activated clay could adsorb tocols during the decolorization. The deodorization process is carried out under high-temperature and high-vacuum conditions. Tocols may be distilled out during this process and then enter the deodorizing distillate with water vapor, leading to the loss of tocols.

#### 2.3.2. Influence of Oil Grades on Tocopherol and Tocotrienol Contents in different Grades Traditional Edible Oils

Five types of traditional edible oils with different grades were investigated. The types of tocols in different grades of edible oil were shown in the Table 4. The types of tocols in different grades of peanut oils were all the same, and it was also same in different grades of rapeseed oils. Both first-grade and third-grade soybean oils had α-T, γ-T and δ-T. However, β-T was detected in some third-grade soybean oils instead of first-grade soybean oils. In terms of cottonseed oils, δ-T was detected in all of first-grade samples but only some of second-grade cottonseed oil samples. All of second-grade rice bran oils included α-T, γ-T, α-T3, γ-T3 and δ-T3. Only α-T and γ-T3 were found in all of first-grade rice bran oils. Some first-grade rice bran oil samples had β-T and δ-T, which were not found in some second-grade rice bran oils. The amount of tocol types in these traditional edible oils were not necessarily related to the grades of edible oils. PCA was used to evaluate the difference between different grades of traditional edible oils, and the results were shown in Figure 3B and Figure S1. Both of the top two principal components (PC1 and PC2) accounted for more than 95.8% of their total variation. The different grades of peanut oils were not well distinguished, and this situation also existed in the other four types of traditional edible oils. Therefore, the contents of tocols in these traditional edible oils were not necessarily related to the grades of edible oils.

The refining degree of a vegetable oil determines its grade. Increasing the degree of oil refining can remove more harmful components in the oil, making the oil grade higher. Meanwhile, the loss of beneficial components in the oil, such as tocopherol, phytosterol, and polyphenol, is also increased [50]. However, according to the results in our study, the tocol content level in the oil could not be directly estimated based on the oil grade. The final content of tocols in the oil is affected not only by the refining degree, but also by a variety of other factors, such as the growing season, climatic conditions during the growing and harvesting of oil crops, seed storage and handling, seed variety, crude oil extraction, blending, and so on [51,52,53]. All the different grades of oil samples used in this study were supplied by different edible oil production factories. The production area of the oil seeds, the oil extraction methods and conditions, the refining conditions, and so on may be different. Although the refining process causes a greater loss of tocols for these low-grade oils, the final tocol content of these low-grade oils is not much different from that of high-grade oils.

## 3. Materials and Methods

### 3.1. Standards and Reagents

Standard tocols (α-, β-, γ-, and δ-tocopherol; α-, β-, γ-, and δ-tocotrienol) were purchased from Sigma-Aldrich (St. Louis, MO, USA) and Calbiochem (Merck, Darmstadt, Germany). Individual stock standard solutions of eight analytes and working standard mixtures were prepared in *n*-hexane, flushed with nitrogen, protected from light, and stored refrigerated (−20 °C). HPLC-grade isopropanol, *n*-hexane, and acetic acid were obtained from Merck (Darmstadt, Germany).

### 3.2. Collection of Traditional Edible Oils

A total of 146 traditional edible oil samples, chosen from nine different kinds of vegetable oils including peanut oils, rice bran oils, corn oils, sunflower oils, cottonseed oils, camellia oils, sesame oils, soybean oils, and rapeseed oils, were all supplied by edible oil production factories. The number of selected first-grade vegetable oil samples is presented in Table 1. Peanut oils, rice bran oils, cottonseed oils, and soybean oils all included two grades and rapeseed oils included four grades. The number of these edible oils is presented in Table 2. All the traditional edible oil samples were stored in the dark and used immediately after opening.

### 3.3. Preparation of New Types of Vegetable Oil

#### 3.3.1. Collection of Raw Oil Seed Samples

Ten types of raw oil seed samples used for the production of new types of vegetable oil were purchased from markets. Plants bearing these oil seeds included yellow horn (*Xanthoceras sorbifolium* Bunge), walnut (*Carya cathayensis* Sarg.), Chinese sumac (*Rhus chinensis* Mill.), sacha inchi (*Plukenetia volubilis* L.), tigernut (*Cyperus esculentus* L.), hazelnut (*Gevuina avellana* Mol), swida wilsoniana (*Swida wilsoniana* (Wanger.) Sojak), suaeda salsa (*Suaeda salsa* (L.) Pall), kenaf (*Hibiscus cannabinus* L.), and eucommia (*Eucommia ulmoides* Oliv.). All the raw oil seed samples were collected immediately after harvest and then stored in a medical freezer at −20 °C and atmospheric pressure in the dark with a 53% humidity until usage.

#### 3.3.2. Extraction of New Types of Vegetable Oil

The extraction of new types of vegetable oil was carried out referring to our published method [54] with some modifications. Raw oil seeds were smashed using a crusher and sieved through a 40-mesh sieve. Smashed raw oil seeds (400–500 g for each sample) and analytical grade *n*-hexane (1000–1200 mL) were mixed in a 2000 mL beaker. Then, the beaker was sealed and held in a water bath (50 °C) with agitation (500 rpm) for 6 h. Subsequently, the mixture was filtered through a filter paper and the filtrate was collected with a Buchner funnel under vacuum. Then, the filtrate was evaporated under vacuum at 55 °C to remove the solvent. Finally, the crude new types of vegetable oil were obtained and stored at −20 °C until usage.

#### 3.3.3. Refining of New Types of Vegetable Oil

Crude vegetable oils contain undesired compounds, some of which (waxes, trace metal ions, ketones, aldehydes, etc.) are harmful to human health. The undesired ingredients should be removed by the refining process of purification, mucilage removal, deacidification, bleaching, and deodorization [55]. The refining process of crude new types of vegetable oil was conducted using the procedure reported by Adhikari et al. [38] with some modifications, and the detailed processes are shown in Figure S2.

### 3.4. Analytical Methods of Tocopherols and Tocotrienols

#### 3.4.1. Sample Preparation

The sample preparation method for the analysis of tocopherols and tocotrienols referred to a previous report [20]. In brief, about 0.5 g of a vegetable oil sample was weighed into a 10 mL volumetric flask. Then, 5 mL of *n*-hexane (HPLC-grade) was added to dissolve the vegetable oil, following by making up the volume with *n*-hexane, shaking and filtrating through a 0.22 µm nylon syringe filter, before injecting into the HPLC system.

#### 3.4.2. Determination of Tocopherols and Tocotrienols

HPLC (Waters 2695, Waters Corp., Milford, MA, USA) equipped with a Waters 2475 fluorescence detector was used. The mobile phase was operated in isocratic mode (*n*-hexane: isopropanol: acetic acid (98.9:0.6:0.5, v:v:v)) with a flow rate of 1 mL/min. The normal-phase chromatographic column (LiChrosorb Si60, 250 × 4.6 mm, 5 µm; Suzhou, China) was held at 40 °C and the injection volume was 10 µL. The emission and excitation wavelengths were set at 330 nm and 290 nm, respectively. The system was controlled by a computer running the Waters-Empower 3 software. The chromatograms of the standards (60 µg/mL), refined sumac fruit oil sample, and sacha inchi oil sample were collected and are presented in Figure S3.

#### 3.4.3. Method Validation

Method validation was performed in terms of the linearity, limit of detection (LOD), limit of quantification (LOQ), recovery, and precision. The linearity range was obtained using the selectively chromatographic conditions and six increasing concentrations of the working standard mixtures. The peak areas of each isomer were used for linear regression analysis. The LOD and LOQ were calculated at a signal-to-noise ratio (S/N) of 3 and 10, respectively. Spiked rice bran oil samples were prepared by adding three levels of the mixed standard solution (including a low, medium, and high spiking level: 5, 10, and 400 mg/kg, respectively) to the oils. The results of the method validation are presented in Table S1.

### 3.5. Data Analysis

The quantification of the tocopherol and tocotrienol content was carried out in triplicate with the external standard method. The chromatographic peaks were carefully integrated and analyzed by the Waters-Empower 3 software (Waters Corp., Milford, MA, USA). ANOVA and Duncan’s multiple range tests (α = 0.05) were conducted with the statistical SPSS software version 22.0. The drawing of the box plots and the principal component analysis (PCA) were performed using Origin 2019 (Origin Lab, Northampton, MA, USA). Heat maps were created using an online website [56].

## 4. Conclusions

The oil type exerted a great impact on the tocol content of traditional edible oils. Distinct types of traditional edible oils appeared to differ in both the types and contents of tocols. Soybean oils, corn oils, and rapeseed oils could be all well distinguished from sunflower oils. Meanwhile, both sunflower oils and cotton seed oils could be well distinguished from camellia oils as well as sesame oils. Among them, rice bran oils contained the most abundant types of tocols (seven types), and the total tocol content of soybean oil was generally higher. Additionally, sacha inchi oil showed a high content of total tocols, at 1780.1 mg/kg, and sumac fruit oil was rich in T3 at 233.0 mg/kg, which provided a new approach to obtaining oils with a high tocol content. The oil refining leads to a loss of tocols in vegetable oil. The higher the degree of refining, the higher the grade of oil and the greater the loss of tocols in vegetable oil. However, the final content of tocols in oil was not necessarily related to the grade of vegetable oil. The final content of tocols in oil was affected by many factors, such as the production area of the oil seeds, the oil extraction methods and conditions, the refining conditions, and so on. This study presented a clear understanding of the relationship between the tocol content and different species as well as grades of edible oils, which would be beneficial for the oil industry and dietary nutrition. Results for new types of oil would provide support for the development of new types of oil seed.

## Figures and Tables

**Table 1:** The compositions and contents (mg/kg) of tocols in nine types of selected first-grade traditional edible oils.

Vegetable Oils	Minimum Content–Maximun Content (mg/kg)										
	Medium Content (mg/kg)										
	α-T	β-T	γ-T	δ-T	α-T3	β-T3	γ-T3	δ-T3	ΣT ^a^	ΣT3 ^b^	Total Tocols
Peanut oil (*n* = 15)	133.2–366.4 200.8	ND ^c^	113.3–314.5 183.2	22.8–38.3 29.6	ND	ND	NQ	ND	282.5–719.3 413.5	NQ	282.5–719.3 413.5
Rice bran oil (*n* = 7)	5.9–503.0 151.8	NQ-5.3 1.0	NQ-190.4 64.4	NQ-84.9 16.1	NQ-275.8 71.9	ND	39.2–231.5 107.6	NQ-22.1 5.4	32.3–621.7 233.2	39.2–504.8 184.9	85.0–1126.5 418.2
Corn oil (*n* = 12)	130.9–235.8 183.9	NQ ^d^	425.4–759.9 623.9	43.5–64.8 52.2	ND	ND	40.8–59.7 53.4	NQ	643.7–1042.5 860.0	40.8–59.7 53.4	684.6–1097.7 913.4
Sunflower oil (*n* = 8)	542.1–870.5 718.4	ND	ND-67.4 21.5	ND-22.0 2.7	ND	ND	NQ	ND	555.3–914.6 743.1	NQ	555.3–914.6 743.1
Cottonseed oil (*n* = 3)	449.3–634.0 553.1	ND	284.5–518.0 402.5	24.0–29.5 26.9	ND-84.5 28.2	ND	NQ	ND	761.1–1118.1 982.5	NQ-84.5 28.2	845.7–1118.1 1010.7
Camellia oil (*n* = 6)	NQ-123.4 59.6	ND	NQ-20.3 6.2	ND	ND	ND	ND	ND	5.9–123.4 65.7	ND	5.9–123.4 65.7
Sesame oil (*n* = 14)	ND	ND	214.0–417.0 320.0	ND	ND	ND	ND	ND	214.0–417.0 320.0	ND	214.0–417.0 320.0
Soybean oil (*n* = 23)	69.6–172.6 115.3	NQ	495.2–799.1 675.5	212.8–305.6 261.6	ND	ND	NQ	NQ	804.5–1246.6 1052.6	NQ	804.5–1246.6 1052.6
Rapeseed oil (*n* = 13)	132.7–248.2 181.0	NQ	303.5–460.7 383.5	26.8–39.9 31.0	ND	ND	ND	ND	498.0–691.7 595.5	ND	498.0–691.7 595.5

^a^ ΣT includes α-, β-, γ-, and δ-T. ^b^ ΣT3 includes α-, β-, γ-, and δ-T3. ^c^ ND: not detected. ^d^ NQ: not quantified.

**Table 2:** The data of tocol contents in different vegetable oils extracted from a literature reference.

Oil Types	Tocols Content (mg/kg)			Reference
	Total Tocols	α-T	γ-T	
Soybean oil	958	/^a^	/	[1]
	999	/	/	[38]
	1026.1	/	/	[20]
	1135.8	/	/	[39]
	1886	/	/	[40]
	/	/	642.7	[20]
	/	/	823.1	[39]
	/	/	390–690	[41]
Sunflower oil	768	/	/	[38]
	/	714.9	/	[4]
	/	604.6	/	[4]
	/	720	/	[4]
Peanut oil	367	/	/	[1]
	259.6	/	/	[40]
Rapeseed oil	649.1	/	/	[20]
	555.2	/	/	[39]
Corn oil	808.3	/	/	[39]
Rice bran oil	867.2	/	/	[39]
Eucommia ulmoides seed oil	1218.24	/	/	[30]
Sumac fruit oil	876.95	/	/	[30]
Kenaf seed oil	531.5	/	/	[42]

**Table 3:** The compositions and contents of tocols in the refined new types of vegetable oil ^k^ (mg/kg).

Vegetable Oil	α-T	β-T	γ-T	δ-T	α-T3	β-T3	γ-T3	δ-T3	ΣT ^l^	ΣT3 ^m^	Total Tocols
yellow horn seed oil	54.2 ± 1.2 ^a^	ND ^n^	463.3 ± 12.3 ^e^	56.2 ± 1.4 ^c^	NQ	ND	43.8 ± 1.0 ^c^	NQ	573.6 ± 14.8 ^d^	43.8 ± 1.0 ^d^	617.4 ± 15.7 ^d^
Walnut oil	74.2 ± 3.1 ^b^	ND	300.8 ± 1.8 ^c^	83.0 ± 2.1 ^d^	NQ	ND	NQ	NQ	458.1 ± 3.9 ^b,c^	ND	458.1 ± 3.9 ^b^
Sumac fruit oil	72.7 ± 1.4 ^b^	70.6 ± 1.1 ^c^	175.6 ± 1.6 ^b^	285.5 ± 5.4 ^e^	NQ	6.6 ± 1.1 ^a^	101.8 ± 3.1 ^e^	124.6 ± 5.2 ^b^	604.3 ± 7.9 ^e^	233.0 ± 9.2 ^g^	837.3 ± 15.8 ^f^
Sacha inchi oil	NQ ^o^	ND	1016.0 ± 5.2 ^h^	708.6 ± 4.1 ^f^	ND	ND	55.5 ± 0.4 ^d^	ND	1724.6 ± 9.2 ^i^	55.5 ± 0.4 ^e^	1780.1 ± 9.6 ^i^
Tigernut oil	173.1 ± 2.8 ^e^	44.7 ± 0.4 ^b^	NQ	ND	ND	ND	ND	ND	217.8 ± 3.2 ^a^	ND	217.8 ± 3.2 ^a^
Hazelnut oil	358.4 ± 2.4 ^g^	ND	102.0 ± 3.1 ^a^	10.8 ± 0.4 ^a^	NQ	ND	29.8 ± 0.5 ^b^	6.1 ± 0.2 ^a^	471.2 ± 5.5 ^c^	36.0 ± 0.3 ^c^	507.2 ± 5.2 ^c^
Swida wilsoniana oil	792.4 ± 3.6 ^h^	ND	NQ	NQ	ND	ND	139.8 ± 1.2 ^g^	ND	792.4 ± 3.6 ^g^	139.8 ± 1.2 ^f^	932.2 ± 4.8 ^g^
Suaeda salsa seed oil	146.4 ± 2.6 ^d^	ND	568.8 ± 16.2 ^f^	30.8 ± 0.7 ^b^	NQ	ND	22.5 ± 0.5 ^a^	NQ	746.1 ± 19.2 ^f^	22.5 ± 0.5 ^b^	768.6 ± 18.6 ^e^
Kenaf seed oil	295.4 ± 4.6 ^f^	ND	425.1 ± 5.1 ^d^	10.2 ± 0.3 ^a^	NQ	ND	20.7 ± 0.7 ^a^	NQ	730.6 ± 9.4 ^f^	20.7 ± 0.7 ^b^	751.3 ± 8.7 ^e^
Eucommia ulmoides seed oil	107.7 ± 2.5 ^c^	25.6 ± 0.7 ^a^	851.1 ± 9.3 ^g^	288.6 ± 6.9 ^e^	3.2 ± 0.2 ^a^	ND	ND	NQ	1273.0 ± 19.3 ^h^	3.2 ± 0.2 ^a^	1276.2 ± 19.4 ^h^

^a–i^ Samples with the same letter in a column are not significantly different (*p* < 0.05). ^k^ Values are mean ± standard deviation of triplicate determinations. ^l^ ΣT includes α-, β-, γ-, and δ-T. ^m^ ΣT3 includes α-, β-, γ-, and δ-T3. ^n^ ND: not detected. ^o^ NQ: not quantified.

**Table 4:** The compositions and contents (mg/kg) of tocols in five types of selected traditional edible oils containing different grades.

Vegetable Oils		Minimum Content-Maximum Content (mg/kg)										
		Medium Content (mg/kg)										
		α-T	β-T	γ-T	δ-T	α-T3	β-T3	γ-T3	δ-T3	ΣT ^a^	ΣT3 ^b^	Total tocols
Peanut oil	G1 ^e^ (*n* = 15)	133.2–366.4 200.8	ND ^c^	113.3–314.5 183.2	22.8–38.3 29.6	ND	ND	ND	ND	282.5–719.3 413.5	ND	282.5–719.3 413.5
	G2 ^f^ (*n* = 4)	183.2–233.9 213.5	NQ ^d^	128.5–219.0 176.4	22.3–33.6 29.9	ND	ND	NQ	ND	334.0–465.4 419.9	NQ	334.0–465.4 419.9
Rice bran oil	G1 (*n* = 7)	5.9–503.0 151.8	ND- 5.3 1.0	ND- 190.4 64.4	ND -84.9 16.1	ND -275.8 71.9	ND	39.2–231.5 107.6	ND- 22.1 5.4	32.3–621.7 233.2	39.2–504.8 184.9	85.0–1126.5 418.2
	G2 (*n* = 4)	483.0–593.3 528.3	ND-11.3 3.8	59.3–86.0 74.5	ND-29.6 13.4	237.0–284.9 258.3	ND-13.2 3.3	216.1–369.3 279.9	21.0–29.8 25.3	542.2–720.3 620.0	490.2–684.0 566.7	1112.7–1279.0 1186.7
Cottonseed oil	G1 (*n* = 3)	449.3–634.0 553.1	ND	284.5–518.0 402.5	24.0–29.5 26.9	ND-84.5 28.2	ND	ND	ND	761.1–1118.1 982.5	ND-84.5 28.2	845.7–1118.1 1010.7
	G2 (*n* = 3)	513.4–631.8 576.4	ND	500.1–533.5 516.8	ND-19.9 13.1	ND	ND	NQ	ND	1033.3–1165.3 1106.2	NQ	1033.3–1165.3 1106.2
Soybean oil	G1 (*n* = 23)	69.6–172.6 115.3	ND	495.2–799.1 675.5	212.8–305.6 261.6	ND	ND	ND	ND	804.5–1246.6 1052.6	ND	804.5–1246.6 1052.6
	G3 ^g^ (*n* = 17)	34.1–149.5 98.8	NQ-12.8 2.1	534.7–824.2 718.3	240.6–443.5 318.9	NQ	ND	NQ	ND	815.6–1322.4 1138.1	NQ	815.6–1322.4 1138.1
Rapeseed oil	G1 (*n* = 13)	132.7–248.2 181.0	ND	303.5–460.7 383.5	26.8–39.9 31.0	ND	ND	ND	ND	498.0–691.7 595.5	ND	498.0–691.7 595.5
	G2 (*n* = 3)	95.0–258.4 169.1	ND	14.6–431.4 265.8	18.5–31.2 25.0	ND	ND	ND	ND	128.1–634.9 459.9	ND	128.1–634.9 459.9
	G3 (*n* = 8)	112.6–299.7 220.6	NQ	361.6–517.0 443.4	30.2–67.8 36.9	ND	ND	NQ	ND	506.7–836.8 701.0	NQ	506.7–836.8 701.0
	G4 ^h^ (*n* = 6)	157.9–283.7 199.1	NQ	417.3–523.0 465.3	30.3–58.2 36.6	ND	ND	NQ	NQ	617.7–837.8 701.0	NQ	617.7–837.8 701.0

^a^ ΣT includes α-, β-, γ-, and δ-T. ^b^ ΣT3 includes α-, β-, γ-, and δ-T3. ^c^ ND: not detected. ^d^ NQ: not quantified. ^e–h^ G1, G2, G3, and G4 represent the first, second, third, and fourth grade of traditional edible oils, respectively.

## Supplementary Materials

Figure S1: Flowchart depicting the refining process of the crude new types of vegetable oil. Figure S2: Representative chromatograms of the working standard mixtures, refined sumac fruit oil sample and refined sacha inchi oil sample. Table S1: Correlation coefficient, recovery, intraday precision, interday precision, LOD and LOQ of the tocols analyzed by HPLC.
